# ERK1/2 Signalling Pathway Regulates Tubulin-Binding Cofactor B Expression and Affects Astrocyte Process Formation after Acute Foetal Alcohol Exposure

**DOI:** 10.3390/brainsci12070813

**Published:** 2022-06-22

**Authors:** Yin Zheng, Jiechao Huo, Mei Yang, Gaoli Zhang, Shanshan Wan, Xiaoqiao Chen, Bingqiu Zhang, Hui Liu

**Affiliations:** 1 Institute of Neuroscience, Chongqing Medical University, Chongqing 400016, China; yangmei503@cqmu.edu.cn (M.Y.); 2020110047@stu.cqmu.edu.cn (X.C.); 2021110072@stu.cqmu.edu.cn (B.Z.); huiliu@cqmu.edu.cn (H.L.); 2 Institute for Viral Hepatitis, Second Affiliated Hospital of Chongqing Medical University, Chongqing 400063, China; 305974@hospital.cqmu.edu.cn; 3 Department of Blood Transfusion, Sichuan Cancer Hospital & Institute, Chengdu 610044, China; 2020110060@stu.cqmu.edu.cn

**Keywords:** tubulin-binding cofactor B, astrocyte processes, microtubules, extracellular signal-regulated protease 1/2 signalling pathway, foetal alcohol spectrum disorders, acute foetal alcohol exposure

## Abstract

Foetal alcohol spectrum disorders (FASDs) are a spectrum of neurological disorders whose neurological symptoms, besides the neuronal damage caused by alcohol, may also be associated with neuroglial damage. Tubulin-binding cofactor B (TBCB) may be involved in the pathogenesis of FASD. To understand the mechanism and provide new insights into the pathogenesis of FASD, acute foetal alcohol exposure model on astrocytes was established and the interference experiments were carried out. First, after alcohol exposure, the nascent astrocyte processes were reduced or lost, accompanied by the absence of TBCB expression and the disruption of microtubules (MTs) in processes. Subsequently, TBCB was silenced with siRNA. It was severely reduced or lost in nascent astrocyte processes, with a dramatic reduction in astrocyte processes, indicating that TBCB plays a vital role in astrocyte process formation. Finally, the regulating mechanism was studied and it was found that the extracellular signal-regulated protease 1/2 (ERK1/2) signalling pathway was one of the main pathways regulating TBCB expression in astrocytes after alcohol injury. In summary, after acute foetal alcohol exposure, the decreased TBCB in nascent astrocyte processes, regulated by the ERK1/2 signalling pathway, was the main factor leading to the disorder of astrocyte process formation, which could contribute to the neurological symptoms of FASD.

## 1. Introduction

Foetal alcohol spectrum disorder (FASD) is an umbrella term that encompasses many congenital abnormalities resulting from prenatal alcohol exposure, including foetal cognitive impairment, behavioural abnormalities, and physical impairment [1]. Foetal alcohol syndrome (FAS) is one of the most severe types of FASD [2]. Many aspects of FASD disorder have been successfully summarised in vitro and in vivo, including morphological and behavioural defects, but the pathogenesis of the disease is still unclear [3,4]. The unawareness of pregnancy or addictive alcoholism in women during pregnancy has led to FASD in recent years.

FASD patients’ brains usually are reduced in size, have prominent abnormalities in shape, with reduced brain growth [5], significantly fewer cells [6], and greater cortical thickness in the frontal lobes [7]. In rodent neonates, the altered morphology of pyramidal neuron dendrites in the medial prefrontal cortex was also found [8]. The structural alterations, especially the medial prefrontal cortex after alcohol exposure, can lead to numerous cognitive and behavioural outcomes [5,6,9]; the medial prefrontal cortex is critical for behaviour through the prefrontal–thalamo–hippocampal circuit [10] and for cognition, including fear, through prefrontal cortex–amygdala–hippocampus circuitry [11]. In recent years, heart rate variability has been used as a biological marker of cognitive changes [12] and the neurovisceral integration model of fear was used to clarify the fear conditioning: the prefrontal cortex, cognitive structures, influence the activity of amygdala and hippocampus, eliciting a neurovisceral fear response through sympathetic and parasympathetic projections that mediate heart-related dynamics [13]. Although the above research showed us some data about FASD, the mechanism of FASD is still not clear. 

Almost all current research on the pathogenesis of FASD has focused on neurons [4]. Alcohol can trigger a robust neurodegenerative response and neuronal apoptosis [14,15] and impact synaptic formation [1,16]. In addition to neurons, astrocytes are also required for synaptic formation [17,18]. Cultured neurons formed fewer weak synapses in the absence of astrocytes than neurons cocultured with astrocytes [16,19]. Indeed, during central nervous system development, astrocytes are more sensitive than neurons to alcohol toxicity [20,21]. Moderate levels of alcohol can delay their growth and maturation [20,22,23]. As the most abundant cells in the nervous system, astrocyte morphology and function alterations must be closely related to the neurological dysfunction of FASD. However, no related studies have been reported thus far. Moreover, almost all of the research focused on chronic alcohol injury [24,25,26,27] causing neurological symptoms of FASD that appeared after birth or even in adolescence. In fact, damage to the foetal nervous system by alcohol occurs during maternal alcohol consumption [28]. However, there have been few reports about acute alcohol injury on nerve cells.

Tubulin-binding cofactor B (TBCB) is a cofactor that helps α-tubulin fold during the de novo synthesis of microtubules [29,30,31,32], which participates in and regulates microtubule assembly [33,34,35], growth, and dynamic stability. Recently, Feltes et al. found an upregulation of TBCB RNA in FASD mice, suggesting a possible relationship between alcohol and TBCB in FASD [29,36]. Interestingly, alcohol was found to cause impaired process formation in astrocytes [20,22,23]. TBCB was shown to affect the growth of axons, the essential processes of neurons, suggesting that TBCB might be involved in the formation of cell processes [37], possibly by acting on the positive end of microtubules, which contributes to the formation of cell processes [30,37]. In summary, there may be some links between TBCB, astrocyte processes, and alcohol in FASD patients and this is worth studying.

Mitogen-activated protein kinase (MAPK) is an evolutionarily conserved major signalling pathway that transmits extracellular stimuli into cells and includes three signalling pathways: extracellular signal-regulated kinase 1/2 (ERK1/2), c-Jun amino-terminal kinase (JNK), and P38 [37]. Previous studies have shown that ethanol can impact the expression of some proteins in neurons [38,39], microglia [40], and other cells [41]. Alcohol, as an exogenous stimulant, is associated with the MAPK pathway [37,38,39,40] and the ERK1/2 signalling pathway is sensitive to alcohol [42]. Therefore, it is worth investigating whether the MAPK signalling pathway regulates TBCB expression in astrocytes after alcohol exposure.

In summary, we hypothesised that some neurological symptoms in FASD patients may be associated with morphological and functional alterations of astrocytes, which may be related to alterations of TBCB expression induced by alcohol exposure, at least partly, and may be regulated by the MAPK signalling pathway. To confirm this speculation, we performed three experiments. First, we studied the relationship between a reduction in TBCB expression and astrocyte morphological alterations after acute foetal alcohol exposure. Then, we silenced the TBCB gene to confirm that the astrocyte morphological alterations were related to the reduction in TBCB expression. Finally, the possible signalling pathway that regulates TBCB expression after acute foetal alcohol exposure was revealed. Our studies will provide new experimental data to fill in the gaps in the pathological mechanisms during the acute injury stage of FASD.

## 2. Materials and Methods

### 2.1. Culture of Primary Astrocytes

Astrocytes were prepared from the cerebral cortex of C57BL/6J mice on postnatal Day 0 [43]. Briefly, newborn mice were sterilised with 75% ethanol and decapitated in sterile condition. Brain tissue was taken out and the meninges were carefully removed. The cerebral cortex was cut into pieces and digested with 0.25% trypsin at 37 °C for 10 min and then digestion was stopped by a mixture of Dulbecco’s modified Eagle’s medium with Ham’s F-12 medium (DMEM/F12; HyClone) supplemented with 10% foetal bovine serum (FBS, Gemini). After centrifugation, the supernatant was discarded and the pellet was resuspended in a mixture of DMEM/F12 supplemented with 10% FBS. Then, the cell suspension was filtered with a 200-mesh filter (Saimike), placed in flasks, and incubated at 37 °C with 5% CO_2_ and 95% air. Cultures were passaged every 4–5 days at least 3 times (G3) to achieve highly pure astrocyte culture [44,45]. The culture medium was replaced every two days. Finally, the cells were transferred to 6-well plates for Western blotting (WB) or reverse transcription real-time PCR (RT–PCR) and in 12-well plates covered with glass slides for immunofluorescence staining (IF). All experiments were performed on astrocytes at passage 3 after being grown for 1 day.

### 2.2. Establishment of the Astrocyte Alcohol Interference Model

Mouse astrocytes were randomly divided into a normal control group (Con) and an acute foetal alcohol exposure group (E). Cells in the control group were cultured conventionally, while cells in the alcohol group were cultured in a conventional medium containing 100 mM alcohol [28,40,46,47,48,49,50,51]. At 1, 6, 12, and 24 h after alcohol exposure, cells in each group were collected for IF (*n* = 6), WB (*n* = 6), and RT–PCR (*n* = 6) detection. 

### 2.3. Small Interfering RNA (siRNA) Transfection

To clarify the effect of the TBCB decrease on the formation of astrocyte processes, a TBCB silencing experiment was carried out. The astrocytes were randomly divided into a blank control group (Con, conventional culture), negative control (NC, transfected with negative control sequence), and a TBCB silencing group (siTBCB, transfected with a TBCB siRNA sequence). TBCB siRNA and scrambled sequences were synthesised by Chongqing Maobai Technology Co. (Chongqing, China). Astrocytes were transfected with TBCB siRNA or negative oligonucleotides in 6-well or 24-well plates for 6 h using the Lipofectamine™ 3000 transfection kit (Invitrogen, USA). Each well of a 6-well plate contained 0.8 × 10^6^ cells, 5 µL siRNA, 3.75 µL Lipofectamine 3000, and 250 μL Opti-MEM (Gibco, Grand Island, NY, USA). Each well of 24-well plates contained 0.6 × 10^5^ cells per well, 1.25 μL siRNA, 0.75 μL Lipofectamine 3000, and 50 μL Opti-MEM [52]. The sequence of TBCB was as follows: forward 5′-GCAUCCAUGUCAUUGACCATT-3′ and reverse 5′-UGGUCAAUGACAUGGAUGCTT-3′. The negative control sequence was forward 5′-UUCUCCGAACGUGUCACGUTT-3′, reverse 5′-ACGUGACGUUCGGAGAATT-3′. Six hours after transfection, the media was replaced with fresh DMEM/F12 supplemented with 10% FBS. At 48 h following transfection, the cells were collected and analysed by RT–PCR (*n* = 6). Then, 72 h after transfection, the cells were collected and analysed by WB (*n* = 6) and IF (*n* = 6). All operations were carried out in strict accordance with the manufacturer’s instructions.

### 2.4. ERK1/2 Signalling Pathway Interference Assay

To confirm whether the ERK1/2 signalling pathway regulated TBCB expression after alcohol exposure, we interfered with it and observed the effects. Astrocytes were randomly divided into three groups: the solvent control group (Con) was cultured in medium containing 2 µL DMSO (Saimike, Chongqing, China) [52]; the ERK1/2 agonist group (TPA) was cultured in medium containing 200 µM TAP (ERK1/2 agonist, CST [52]) dissolved in 2 µL DMSO; and the ERK1/2 inhibitor group (U0126) was cultured in medium containing 10 mM U0126 (ERK1/2 inhibitor, Selleck, Shanghai, China [52]) dissolved in 2 µL DMSO. After 1 h, the medium containing the treatments was removed and complete fresh medium was added. After 12 h, the three groups of cells were collected separately and analysed by WB (*n* = 6).

To test whether the alcohol-induced TBCB alterations were related to the ERK1/2 pathway, we divided the astrocytes into three groups and pretreated them as described above. After 1 h, the medium containing the treatments was removed, complete fresh medium was added to the solvent control group (Con) and complete fresh medium containing 100 mM alcohol was added to the agonist and inhibitor groups. After 12 h, all of the cells were collected and detected by WB (*n* = 6).

### 2.5. Western Blotting

The harvested astrocytes were lysed on ice in RIPA buffer containing 1% PMSF (Beyotime, Guangzhou, China) and the total protein concentration was measured with a BCA protein assay kit (Beyotime, Shanghai, China). After dilution in the sample loading buffer, 20 µg of protein was added to each lane. The proteins were then separated on a 10% SDS–PAGE gel and transferred to a 0.2 μm polyvinylidene difluoride (PVDF) membrane (Millipore, Bedford, MA, USA). The membranes were blocked with blocking buffer (Beyotime, Shanghai, China) at room temperature for 30 min. Then, they were probed with the properly diluted primary antibodies followed by HRP-labelled anti-mouse or anti-rabbit IgG secondary antibody (ZB-2305 or ZB2301, ZSGBBIO, Beijing, China). They were visualised by Western Bright ECL (Advansta, San Jose, CA, USA) and imaged using a Western blotting detection system (Bio-Rad, Hercules, CA, USA) or X-ray film. Each sample was repeated 3 times and each blot was imaged 3 times. Then, the densities of the bands in each image were quantified 3 times by Quantity-One software. The value of the target protein was normalised to the value of the housekeeping protein from the same sample within the same blot. Then, all of the corresponding values from the different groups were statistically analysed by GraphPad Prism 6.0 software (GraphPad Software, San Diego, CA, USA) [53].

The locations of all proteins detected by the antibodies used in WB are shown in the full-length blots in Supplementary S1. The primary antibodies used in WB were as follows: anti-TBCB (1:500, A13248, ABclonal, Wuhan, China), anti-p38 (1:1000, #8690, Cell Signalling Technology, Danvers, MA, USA), anti-pp38 (1:800, #4511, Cell Signalling Technology, Danvers, MA, USA), anti-JNK (1:1000, #9252, Cell Signalling Technology, Danvers, MA, USA), anti-p-JNK (1:1000, #4668, Cell Signalling Technology, Danvers, MA, USA), anti-β-actin (1:5000, 20536-1-AP, Proteintech, Wuhan, China), anti-α-tubulin (1:5000, GTX628802, GeneTex, Irvine, CA, USA), anti-β-T (1:5000, TA503129, OriGene, Rockville, MD, USA), anti-ERK1/2 (1:1000, #4695, Cell Signalling Technology, Danvers, MA, USA), anti-pERK1/2 (1:1000, #4370, Cell Signalling Technology, Danvers, MA, USA), and anti-GAPDH (1:5000, 60004-1-lg, Proteintech, Wuhan, China).

### 2.6. Reverse Transcription Real-Time PCR

Total RNA was extracted from harvested astrocytes (*n* = 6) by using RANiso plus (#9108, TaKaRa, Beijing, China) [54] and the concentration of RNA was measured by spectrophotometer. A total of 1 µg of RNA was reverse transcribed to generate cDNA using the PrimeScript™ II 1st Strand cDNA Synthesis Kit (TaKaRa, Beijing, China). Messenger expression of TBCB as a housekeeping gene was assessed by real-time PCR. PCR amplification was performed using a T100 thermal cycler (BIO-RAD) and Premix Taq™ (TaKaRa, Beijing, China). The PCR mixture consisted of 1 μL of each primer, 25 μL of Premix Taq, and 1 μL of cDNA in a final volume of 50 μL. The PCR conditions were denaturation at 94 °C for 3 min, followed by 34 cycles of denaturation at 94 °C for 30 s, annealing at 55 °C for 30 s, and extension at 72 °C for 30 s. All qPCRs were run on a CFX96 real-time system (Bio-Rad). The 2^−ΔΔCt^ method was used to calculate the RNA or miRNA level fold change compared to the control samples [52]. The primer sequences (5′- > 3′) were as follows: TBCB, forward ATGGAGCAGACGACAAGTTCT, reverse CCGTCATCCACAGGATAGGAG, product size (77 bp); β-actin (control), forward CAGCCTTCCTTCTTGGGTA, reverse TTTACGGATGTCAACGTCACAC, product size (87 bp). All operations were carried out in strict accordance with the manufacturer’s instructions.

### 2.7. Immunofluorescence Staining

The astrocytes were fixed in −20° precooled acetone and methanol (1:1) for 5 min and then blocked with 5% bovine serum albumin (BSA) at room temperature for 30 min. The astrocytes were probed with the indicated primary antibodies (anti-TBCB, 1:50, A13248, ABclonal, China; anti-TBCB, 1:250, sc-377139, Santa Cruz, USA; anti-α- tubulin, 1:5000, GTX628802, GeneTex, Irvine, California, USA) properly diluted at 4° overnight. Then, the cells were incubated with secondary antibodies (FITC goat anti-rabbit IgG, 1:200, E031220-01, EARTH, China; Cy3 goat anti-mouse IgG, 1:200, Abbkine, Wuhan, China) and stained with DAPI (C1005, Beyotime, Shanghai, China). Subsequently, the cells were mounted in Fluorescence Mounting Medium (ab104135, Abcam, Cambridge, UK) and sealed with nail polish. Images were obtained by confocal laser scanning microscopy (Leica DMI8, Germany) and the intensity of the fluorescence was analysed by ImageJ (1.53 c) software (*n* = 6) [53,55]. The astrocyte processes in the high magnification images were also counted by ImageJ (1.53 c) software (approximately 50 cells were counted in each group). IF was mainly used to observe the changes in cell morphology and protein distribution in this study.

### 2.8. Statistical Analysis

Statistical analyses were performed, and the corresponding graphs were drawn using GraphPad Prism 6.0 software (GraphPad Software, San Diego, CA, USA). All experimental data were expressed as means ± standard deviation (SD). Differences between the treatment group and control group were compared using analysis of a two-sample unpaired *t*-test. All reported p values were two-sided and a value of *p* < 0.05 was considered statistically significant. All the data presented in the manuscript is presented in Table 1.

## 3. Results

### 3.1. Alcohol Inhibited the Formation of Astrocyte Processes and TBCB Expression in Nascent Processes

**Figure 1:**
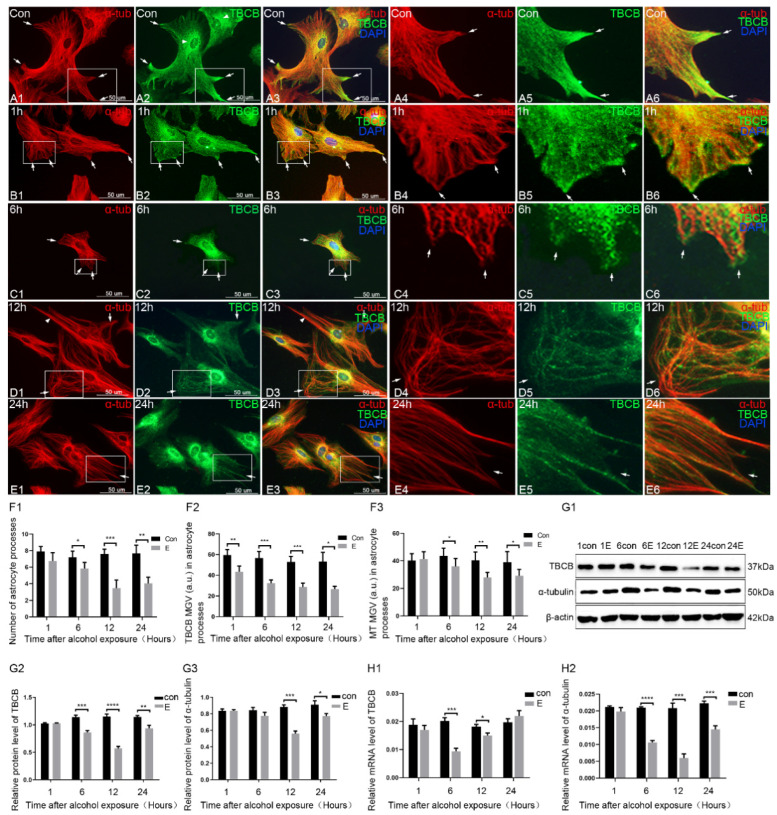
Disorder of astrocyte formation and decreased expression of TBCB after 1, 6, 12, and 24 h of acute foetal alcohol exposure. (**A**–**E**) Immunofluorescence showing changes in the expression of α-tubulin (red signal) and TBCB (green signal) in astrocytes after 1, 6, 12, and 24 h of acute foetal alcohol exposure. Panels A-E 4-6 are magnified images from the square area of Panels A-E 1-3. (**F**) The number of astrocyte processes and mean grey value (MGV) of TBCB and MT in astrocyte processes (MGV = integrated density/area) after 1, 6, 12, and 24 h of acute foetal alcohol exposure. (**G**) Western blot and (**H**) RT–PCR analysis of the protein and mRNA levels of TBCB and α-tubulin after 1, 6, 12, and 24 h of acute foetal alcohol exposure (*n* = 6, *p* < 0.05). *: *p* < 0.05, **: *p* < 0.01, ***: *p* < 0.001, ****: *p* < 0.0001.

We established an acute foetal alcohol exposure astrocyte model to detect the morphological changes in astrocytes and to reveal the possible link between the change in astrocytes and TBCB. The basic morphology, number, status, and structure of the astrocytes had no noticeable difference except for an increased cell volume and the elongating processes at different time points in the control group (Figure S2 and Figure 1F), so only the IF images at 12 h are shown in Figure 1 as the control group (Figure 1A).

In the control group, the astrocytes grew well, with flat and plump bodies and plentiful processes (Figure 1A1–A3). Most of the MTs in the cells were arranged in a linear, filamentous, and radial pattern from the MT organising centre to the edge of the cell cortex, with a uniform and dense distribution and they were especially abundant in the nascent processes (Figure 1A1,A4, arrows). TBCB was distributed with a diffuse punctiform pattern in the cytoplasm or along the MTs. It was highly abundant in the nascent processes (Figure 1A2, arrows), suggesting that TBCB might have an important role in the formation of the nascent processes of astrocytes. It was also rich around the MT organising centre (Figure 1A2, arrowheads).

Compared with the control group, in the alcohol exposure group, the astrocyte cell body gradually shrank and collapsed and the processes were significantly reduced (Figure 1B–E,F1). The sharp tips of newly generated processes became blunt or began to retract (Figure 1B, arrows) at 1 h after treatment with alcohol. The processes very clearly retracted (Figure 1C, arrows and Figure 1F1) at 6 h and few new processes were formed, with the cells collapsing significantly after 12 h (Figure 1D, arrows and Figure 1F1). The density of MT was gradually reduced in processes (Figure 1F3). The MTs were disordered or intertwined into bundles (Figure 1D,E, arrowheads) and most of the positive ends became curly (Figure 1D, arrows), especially in the nascent processes, suggesting a growth disorder at the positive end of the MTs. Along with the retraction and disappearance of the astrocyte processes, TBCB, which was originally abundant in the processes, was also significantly weakened or it disappeared (Figure 1F2), suggesting a close relationship between TBCB and astrocyte processes (Figure 1C2,C3, arrows). WB (Figure 1G) and PCR (Figure 1H) showed that TBCB and α-tubulin expression decreased at 6 h, reaching a trough at 12 h and still lower than the control group at 24 h, suggesting that alcohol could cause a decrease in TBCB and α-tubulin expression.

The above results made it clear that alcohol could impede the formation of astrocyte processes and TBCB expression in these processes. It also suggested that there should be some links between the impaired formation of nascent astrocyte processes and the loss of TBCB expression. To verify this speculation, we used siRNA to silence TBCB expression in astrocytes.

### 3.2. Silencing TBCB Led to Inhibition of Astrocyte Process Formation

**Figure 2:**
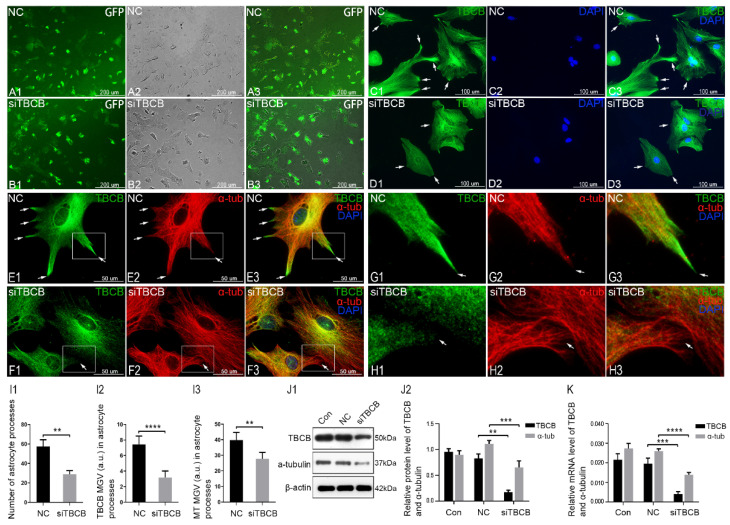
Inhibition of astrocyte process formation after TBCB was silenced. (**A**,**B**) Immunofluorescence shows that the positive transfection efficiency with TBCB siRNA with green fluorescence was 80%. (**C**,**D**) In lower magnification images, immunofluorescence shows a change in TBCB expression at nascent astrocyte processes (arrows) after TBCB silencing. (**E**–**H**) Immunofluorescence shows changes in the expression of TBCB (green signal) and α-tubulin (red signal) in astrocytes in both the control group (**E**,**G**) and the TBCB-silenced group (**F**,**H**). Panels G and H are magnified images from the square area of Panels E and F. (**I**) The number of astrocyte processes and mean grey value (MGV) of TBCB and MT in astrocyte processes (MGV = integrated density/area) after 1, 6, 12, and 24 h of acute foetal alcohol exposure. (**J**) Western blot and (**K**) RT–PCR analyses of the protein and mRNA levels of TBCB and α-tubulin after TBCB was silenced (*n* = 6, *p* < 0.05). NC: negative control group; siTBCB: TBCB siRNA group. **: *p* < 0.01, ***: *p* < 0.001, ****: *p* < 0.0001.

To clarify the relationship between the reduction in TBCB and the inhibition of astrocyte process formation, we performed TBCB silencing experiments with siRNA. In this experiment, the transfection efficiency of TBCB siRNA was higher than 80% and the positive astrocytes showed green fluorescence (Figure 2A,B1–B3). In the silencing group, the mRNA and protein expression of TBCB was significantly downregulated, as shown by PCR (Figure 2K) and WB (Figure 2J), which proved that the silencing effect of TBCB was good.

After silencing, TBCB expression was obviously reduced in astrocytes (Figure 2D,F1,F3), most notably in the nascent processes (Figure 2H1,H3,I2), where originally TBCB expression was extremely abundant (Figure 2C,E,G1,G3, arrows), along with the loss of most nascent cell processes (Figure 2D,F1,F3,I1). These results indicated that the absence of TBCB in the nascent processes of astrocytes led to disordered astrocyte process formation. In addition, it was accompanied by a severe reduction in intracellular MT density (Figure 2F2), most obviously in the nascent processes where MTs were reduced (Figure 2H2,I3), disordered, and intertwined with curly plus ends (Figure 2H2, arrows), suggesting that the severe disorder or dysfunction at the positive end of MTs and TBCB in astrocyte processes was related to the formation and growth of the plus ends of MTs.

This experiment confirmed that TBCB was involved in the formation of astrocyte processes by regulating the plus end of MT. Thus, combined with the first part’s results, it was revealed that the disorder of astrocyte process formation after alcohol exposure was associated with a reduction in TBCB expression in the processes caused by alcohol through regulating the MT plus end. These results provide an essential mechanism for understanding the pathogenesis of FASD and the signalling pathway involved in this process is worth studying.

### 3.3. ERK1/2 Signalling Pathway Regulated TBCB Expression after Acute Foetal Alcohol Exposure in Astrocytes

**Figure 3:**
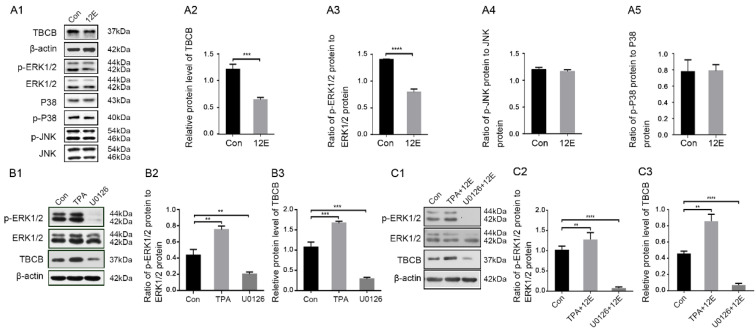
Changes in protein levels in the TBCB and ERK1/2 signalling pathways after alcohol exposure and the protein levels of TBCB and α-tubulin after interfering with the ERK1/2 signalling pathway. (**A**) Western blot analysis of phosphorylation levels and relative quantitative analysis of TBCB and MAPK (ERK1/2, p38, JNK) signalling pathways in astrocytes after 12 h of alcohol exposure (*n* = 6, *p* < 0.05). (**B**) Western blot analysis of the p-ERK1/2 to ERK1/2 ratio and protein levels of TBCB and α-tubulin in astrocytes after pretreatment with the ERK1/2-specific inhibitor U0126 or agonist TPA (*n* = 6, *p* < 0.05). (**C**) Western blot analysis of the p-ERK1/2/ERK1/2 ratio and protein levels of TBCB and α-tubulin in astrocytes after pretreatment with U0126 or TPA before alcohol exposure (*n* = 6, *p* < 0.05). Con: control group; 12E: ethanol or alcohol exposure group; TAP: ERK1/2 agonist group; U0126: ERK1/2 inhibitor group; TPA + 12E: ERK1/2 agonist and ethanol exposure group; U0126 + 12E: ERK1/2 inhibitor and ethanol exposure group. **: *p* < 0.01; ***: *p* < 0.001, ****: *p* < 0.0001.

Compared with the control group, TBCB expression was reduced at 12 h after alcohol exposure (Figure 3A1,A2, *p* < 0.001). In the MAPK signalling pathway as shown by WB, *p*-ERK1/2 expression was significantly reduced (Figure 3A1,A3, *p* < 0.0001), while the changes in p-JNK and p-P38 proteins were not noticeable (Figure 3A1,A4,A5, *p* > 0.05). This result suggested that the ERK1/2 signalling pathway may regulate TBCB expression after alcohol exposure.

To verify that the ERK1/2 signalling pathway is one of the main signalling pathways regulating TBCB expression in astrocytes, the cells were pretreated with the ERK1/2-specific inhibitor U0126 and agonist TPA for 1 h. Compared with the control group, in the agonist pretreatment group, p-ERK1/2 expression was significantly upregulated (Figure 3B1,B2, *p* < 0.01), accompanied by a significant enhancement of TBCB (Figure 3B1,B3, *p* < 0.001). In the inhibitor pretreatment group, p-ERK1/2 expression was significantly decreased (Figure 3B1,B2, *p* < 0.01), accompanied by a significant decrease in TBCB (Figure 3B1,B3, *p* < 0.001). These results confirmed that the ERK1/2 signalling pathway regulates the expression of TBCB in astrocytes.

To further confirm that the ERK1/2 pathway was related to the regulation of TBCB expression in astrocytes after alcohol exposure, astrocytes were pretreated with U0126 or TPA for 1 h and then exposed to alcohol for 12 h, the time point at which the reduction in TBCB was most obvious. Similarly, TBCB expression was significantly lower in the inhibition pretreatment group and higher in the agonist pretreatment group (Figure 3C1–C3, *p* < 0.05) compared with the single alcohol exposure group. These data further demonstrated that the ERK1/2 signalling pathway is one of the main pathways regulating TBCB expression in astrocytes after alcohol injury.

## 4. Discussion

In order to clarify the role of TBCB in the morphological changes of astrocytes after acute foetal alcohol exposure, we carried out the above experiments. In this study, we confirmed that decreased TBCB was one of the critical factors for the formation and growth disorder of astrocyte processes after chronic alcohol exposure; TBCB, which could be regulated by the ERK signalling pathway, regulated the growth of MT plus-ends through binding with EB1 and EB3; thus, regulating the formation and growth of astrocyte processes. In addition, our study also showed the following interesting findings.

### 4.1. Possible Functions of TBCB

Previous studies have demonstrated that TBCB is involved in the de novo synthesis of MT [29,30,31,32]. MTs are polar structures of the cytoskeleton formed by the head-to-tail association of αβ heterodimers. The heterodimers bind to protofilaments associated laterally, forming a hollow cylindrical wall, the microtubule [30,56,57]. MTs have different polymerisation rates at their two ends. The slower-growing minus end binds to the perinuclear centrosome-based MT-organising centre and the faster-growing plus end radiates to the cell edges [57,58]. In general, the plus end undergoes cycles of rapid growth and disassembly, known as dynamic instability, which allows microtubules to reorganise rapidly and differentiate spatially and temporally in accordance with the cell context, generate pushing and pulling forces, searching the cell’s three-dimensional space and leading to cell asymmetry [59].

TBCB was reported to play an essential role in the dynamics of MT in early oocytes [60] and microglia [61]. It was also found to increase at the end of the axon, the essential neural protrusions, characterised by an assembled MT plus end [37]. These data suggest that TBCB might be involved in the MT plus end’s dynamic changes and be related to the cell processes. In our experiments, TBCB was highly expressed in the nascent processes of astrocytes and was coexpressed with the plus end of MT. Moreover, silencing TBCB reduced the expression of TBCB in the processes of astrocytes, accompanied by disordered formation and growth of astrocyte processes, revealing an essential role of TBCB in the formation and growth of astrocyte processes by regulating the plus end of MT. These results may provide new ideas for the study of neurological diseases.

In our experiment, in addition to its diffuse high expression in the nascent process, TBCB could distribute along the MTs and was abundant in the perinuclear area and MT organising centre. This result was similar to Vadlamudi RK’s results. They observed colocalisation between TBCB and tubulin during interphase and mitosis, in which TBCB was associated with staining of the mitotic spindle [62]. These results suggested that TBCB might be involved in MT growth at the minus-end and cell division.

The diffuse expression of TBCB decreased sharply after TBCB was silenced (Figure 2B). However, TBCB arranged along preexisting MTs was still visible, with these MTs maintaining their original structure (Figure 2D,F1), suggesting that TBCB might be associated with the stability of the preexisting MT. Although some functions of TBCB were found, it remains a mystery that needs more study.

### 4.2. Regulating Mechanism of TBCB

We found that ERK1/2 and the PAK1 pathway were inhibited in alcohol-exposed astrocytes. These results were similar to previous studies on neurons of the hippocampus [38] and microglia [40], in which alcohol also caused a decrease in p-ERK1/2 expression. To confirm that the ERK1/2 signalling pathway regulates TBCB expression in normal astrocytes and in astrocytes after alcohol exposure, we detected TBCB expression after treatment with an inhibitor or agonist of ERK1/2. As expected, in normal and alcohol-exposed astrocytes, TBCB expression was regulated by the ERK1/2 signalling pathway. Therefore, after alcohol exposure, inhibiting the ERK1/2 pathway caused by alcohol led to the downregulation of TBCB expression, resulting in disordered MT plus end growth and finally disrupting the astrocyte process formation. This study provides a new view and experimental basis for revealing the neurological symptoms caused by astrocyte injury in FASD.

### 4.3. Astrocytes and FASD

The upregulated TBCB mRNA expression found by Feltes et al. when analysing transcriptomic data from mice suffering from FASD [36], seemed to conflict with our experimental results, which showed downregulation of TBCB protein expression after alcohol exposure in astrocytes. The upregulated mRNA data came from the brain of newborn or one-month old mice, which suffered alcohol injury when they were foetuses by intraperitoneal injection of alcohol into the mother mice. However, the nervous system injury of the foetal mice caused by alcohol occurs immediately after the mother mice are subjected to intraperitoneal injection. Studies have shown that alcohol can immediately cause the loss of proteins [63], severe DNA damage [64], significant ultrastructure injury [65], and so on from 1 to 48 h after alcohol exposure. These results coincided with ours and proved that the damage to cell structures and the loss of protein, such as TBCB in astrocytes, occurred immediately after alcohol exposure. Then, the cells that suffered from alcohol toxicity would upregulate various mRNAs to compensate for the lost proteins to help the organism recover over time after alcohol injury, unless the cell damage was too severe to recover from. Therefore, the upregulated TBCB mRNA in newborn or one-month old mice [29,30] compensated for the loss of TBCB caused by alcohol toxicity during the foetal stage. These results also highlighted the important role of TBCB in the development of the nervous system.

Astrocyte processes are involved in most astrocyte functions. They contact nodes of Ranvier in brain white matter to regulate nerve conduction [66], contact with blood vessels to control blood flow, and form gap junctions with neighbouring astrocytes [67]. Particularly, in the brain the processes envelop and cover about 60% of synapses, called perisynaptic processes and form the astroglial synaptic cradle, which contributes to synaptogenesis, synaptic maturation, synaptic maintenance, and synaptic extinction [18,68].

The first postnatal week in rodents is known as the ‘brain growth spurt’ period, also called the synaptogenesis period, with the marked formation of synapses and neuronal networks [1,15]. Our experiments were performed during this period and we found that a decrease in TBCB expression in astrocyte processes was one of the leading causes of the disorder of astrocyte process formation induced by alcohol toxicity. The absence or disappearance of perisynaptic processes, due to the formation disordered of astrocyte process, would certainly lead to the impairments of neuronal synapse, such as formation, substrate transmission, energy metabolism [18], glutamate metabolism [19,69], and so on. These finally could cause neurological symptoms. Combined with our experimental results, these neurological symptoms could be caused by disordered astrocyte process formation because of TBCB loss after alcohol exposure, providing a new idea to study the pathogenic mechanism of FASD.

Although we believe that astrocyte injury can cause the neurological impairment in FASD, we still cannot address which symptom is caused by neuronal injury and which by astrocytic injury. As well, in this study, we confirmed that TBCB could regulate the formation of astrocyte processes; however, the mechanism is still unclear. Future research should explore how TBCB regulates the astrocyte process formation, maybe through regulating the MT plus-end by interacting with some other factors.

## 5. Conclusions 

Our results show that acute foetal alcohol exposure of astrocytes leads to a decrease in TBCB expression, which first affects the positive end of MT and then leads to disorders in the formation, growth, and development of astrocyte processes. The ERK1/2 signalling pathway positively regulates TBCB expression; thus, affecting the growth of astrocytes. Our findings provide new insights into the mechanism of alcohol on nerve cells and provides an experimental and theoretical basis for exploring the pathogenesis of FASD.

## Figures and Tables

**Table 1:** Statistical tests used in the study.

Corresponding Figures	Prerequisites	Main Test
Figure 1 F1	Single factor, unpaired	Unpaired *t* test, 1 h group, t(10) = 1.922, *p* = 0.1029 6 h group, t(10) = 2.523, *p* = 0.0451 12 h group, t(10) = 7.421, *p* = 0.0003 24 h group, t(10) = 5.733, *p* = 0.0012
Figure 1 F2	Single factor, unpaired	Unpaired *t* test, 1 h group, t(10) = 5.200, *p* = 0.0004 6 h group, t(10) = 8.538, *p* < 0.0001 12 h group, t(10) = 8.906, *p* < 0.0001 24 h group, t(10) = 7.085, *p* < 0.0001
Figure 1 F3	Single factor, unpaired	Unpaired *t* test, 1 h group, t(10) = 0.3504, *p* = 0.7333 6 h group, t(10) = 2.326, *p* = 0.0424 12 h group, t(10) = 4.234, *p* = 0.0017 24 h group, t(10) = 2.651, *p* = 0.0243
Figure 1 G2	Single factor, unpaired	Unpaired *t* test, 1 h group, t(10) = 0.4869, *p* = 0.6518 6 h group, t(10) = 10.23, *p* = 0.0005 12 h group, t(10) = 17.09, *p* < 0.0001 24 h group, t(10) = 5.931, *p* = 0.0040
Figure 1 G3	Single factor, unpaired	Unpaired *t* test, 1 h group, t(10) = 0.4869, *p* = 0.6518 6 h group, t(10) = 2.177, *p* = 0.0950 12 h group, t(10) = 12.23, *p* = 0.0003 24 h group, t(10) = 4.363, *p* = 0.0120
Figure 1 H1	Single factor, unpaired	Unpaired *t* test, 1 h group, t(10) = 1.117, *p* = 0.3265 6 h group, t(10) = 11.57, *p* = 0.0003 12 h group, t(10) = 4.453, *p* = 0.0112 24 h group, t(10) = 1.665, *p* = 0.1713
Figure 1 H2	Single factor, unpaired	Unpaired *t* test, 1 h group, t(10) = 1.896, *p* = 0.1309 6 h group, t(10) = 28.15, *p* < 0.0001 12 h group, t(10) = 13.14, *p* = 0.0002 24 h group, t(10) = 10.48, *p* = 0.0005
Figure 2 I1	Single factor, unpaired	Unpaired *t* test, t(10) = 6.148, *p* = 0.0008
Figure 2 I2	Single factor, unpaired	Unpaired *t* test, t(10) = 8.803, *p* < 0.0001
Figure 2 I3	Single factor, unpaired	Unpaired *t* test, t(10) = 4.585, *p* = 0.0010
Figure 2 J	Single factor, unpaired	Unpaired *t* test,
		TBCB: t(10) = 6.911, *p* < 0.0001
		α-tubulin: t(10) = 9.021, *p* = 0.0001
Figure 2 K	Single factor, unpaired	Unpaired *t* test,
		TBCB: t(10) = 8.661, *p* = 0.0010 α-tubulin: t(10) = 12.73, *p* = 0.0002
Figure 3 A2	Single factor, unpaired	Unpaired *t* test, t(10) = 9.365, *p* = 0.0007
Figure 3 A3	Single factor, unpaired	Unpaired *t* test, t(10) = 18.61, *p* < 0.0001
Figure 3 A4	Single factor, unpaired	Unpaired *t* test, t(10) = 1.169, *p* = 0.3072
Figure 3 A5	Single factor, unpaired	Unpaired *t* test, t(10) = 2.412, *p* = 0.2318
Figure 3 B2	Single factor, unpaired	Unpaired *t* test,
		Con vs. TPA: t(10) = 6.544, *p* = 0.0028 Con vs. U0126: t(10) = 0.0059, *p* = 0.0059
Figure 3 B3	Single factor, unpaired	Unpaired *t* test,
		Con vs. TPA: t(10) = 7.650, *p* = 0.0016 Con vs. U0126: t(10) = 10.30, *p* = 0.0005
Figure 3 C2	Single factor, unpaired	Unpaired *t* test,
		Con vs. TPA: t(10) = 3.278, *p* = 0.0083 Con vs. U0126: t(10) = 23.99, *p* < 0.0001
Figure 3 C3	Single factor, unpaired	Unpaired *t* test,
		Con vs. TPA: t(10) = 7.623, *p* = 0.0016 Con vs. U0126: t(10) = 18.46, *p* < 0.0001

## Data Availability

The data presented in this study are available on request from the corresponding author.

## Supplementary Materials

The following are available online at https://www.mdpi.com/article/10.3390/brainsci12070813/s1.
