# Supplementary material for: ERK1/2 Signalling Pathway Regulates Tubulin-Binding Cofactor B Expression and Affects Astrocyte Process Formation after Acute Foetal Alcohol Exposure
Source: Brain Sci. 2022 Jun 22;12(7):813. doi: 10.3390/brainsci12070813 (PMC9312805; doi:10.3390/brainsci12070813)
Supplement: Supplementary file 1 [file brainsci-12-00813-s001.zip › Suppl.S1 legend.pdf]

Supplementary S1. Astrocytes identify by GFAP and localizations of all the proteins in this study shown in full-length Western Blot image. (A) Astrocytes identify by GFAP. **(B)** Anti-TBCB (1:500, A13248, ABclonal, China), **(C)** Anti-α-T(1：5000，GTX628802, GeneTex, USA), **(D)** Anti-β-actin (1:5000,20536-1-AP, Proteintech, China), **(E)** Anti-β-T (1:5000，TA503129, OriGene, USA), **(F)** Anti-GAPDH (1:5000, 60004-1-lg, Proteintech, China), **(G)** Anti-ERK (1:1000, #4695, Cell Signaling Technology, USA), **(H)** Anti-pERK (1:1000, #4370, Cell Signaling Technology, USA), **I)** Anti-p38 (1:1000, #8690, Cell Signaling Technology, USA), **(J)** Anti-pp38 (1:800, #4511, Cell Signaling Technology, USA), **(K)** Anti-JNK (1:1000, #9252, Cell Signaling Technology, USA), **(L)** Anti-p-JNK (1:1000, #4668, Cell Signaling Technology, USA).
