# Supplementary material for: ERK1/2 Signalling Pathway Regulates Tubulin-Binding Cofactor B Expression and Affects Astrocyte Process Formation after Acute Foetal Alcohol Exposure
Source: Brain Sci. 2022 Jun 22;12(7):813. doi: 10.3390/brainsci12070813 (PMC9312805; doi:10.3390/brainsci12070813)
Supplement: Supplementary file 1 [file brainsci-12-00813-s001.zip › Suppl.S2 legend.pdf]

Supplementary S2 No noticeable difference in morphology, number, status and structure of astrocytes at 1 h, 6 h, 12 h, 24 h in control group. (A-D) Immunofluorescence showed that α-tubulin (red signal) was co-expressed with TBCB (green signal) at 1 h, 6 h, 12 h and 24 h in control group. **(E)** The number of astrocyte processes and mean gray value (MGV) of TBCB and MT in astrocytes and its processes (MGV = Integrated Density/Area) after 1 h, 6 h, 12 h and 24 h acute alcohol exposure. Astrocytes' status in all-time points was good with many nascent processes in which TBCB were highly expressed. Astrocyte processes numbers and IF density of TBCB and α-T showed no noticeable difference.
