# Supplementary figures and images for: ERK1/2 Signalling Pathway Regulates Tubulin-Binding Cofactor B Expression and Affects Astrocyte Process Formation after Acute Foetal Alcohol Exposure
Source: Brain Sci. 2022 Jun 22;12(7):813. doi: 10.3390/brainsci12070813 (PMC9312805; doi:10.3390/brainsci12070813)

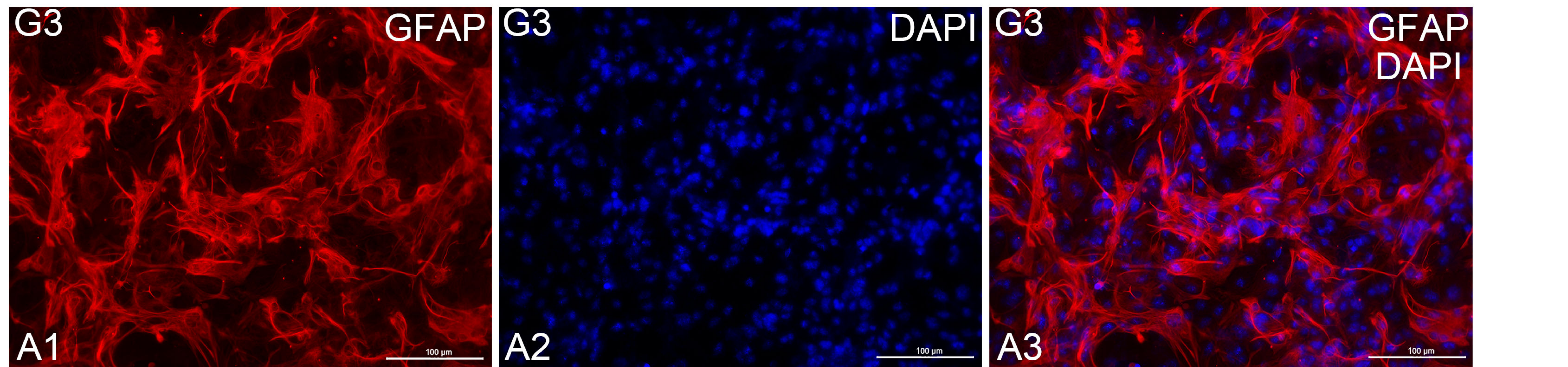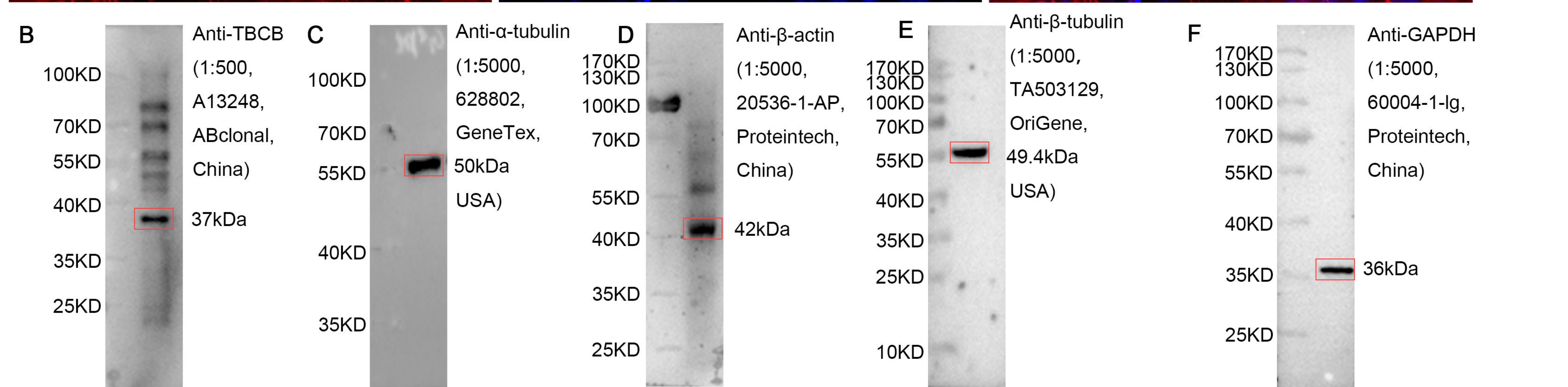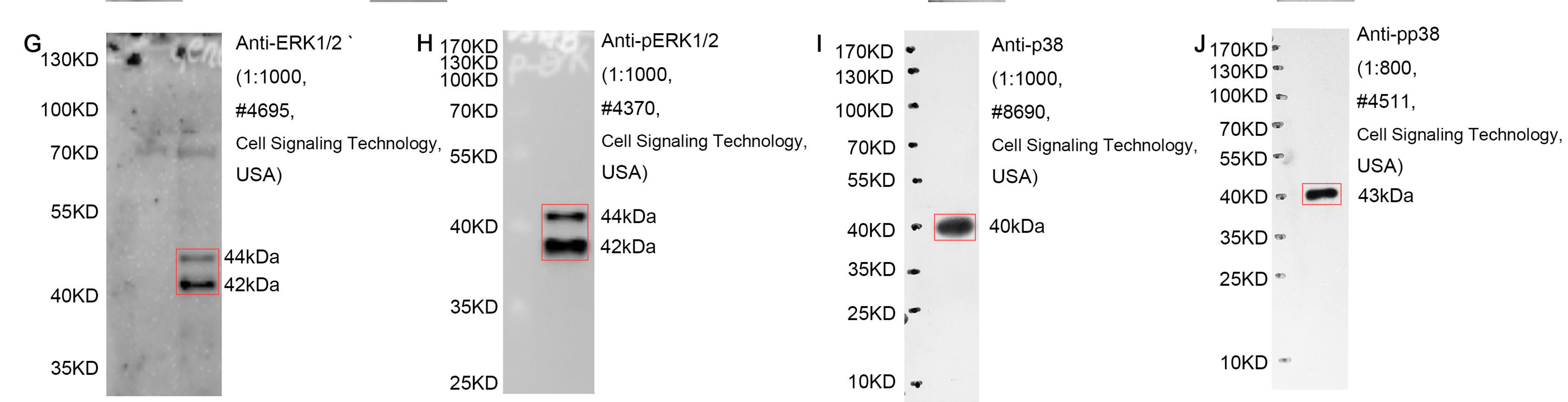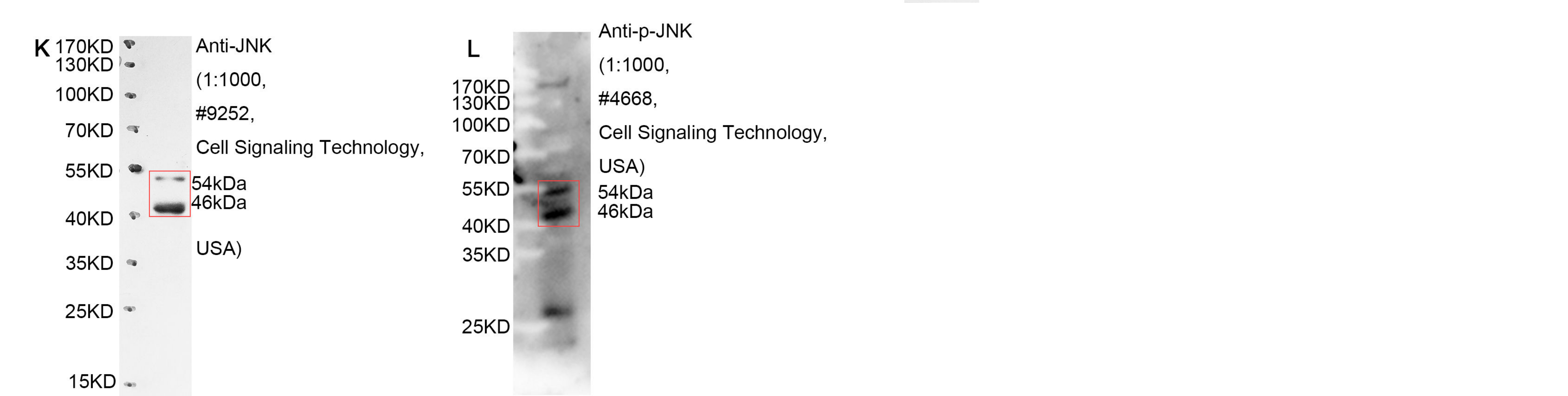

Supplement: Supplementary file 1 [file brainsci-12-00813-s001.zip › Suppl.S1-S2.pdf]

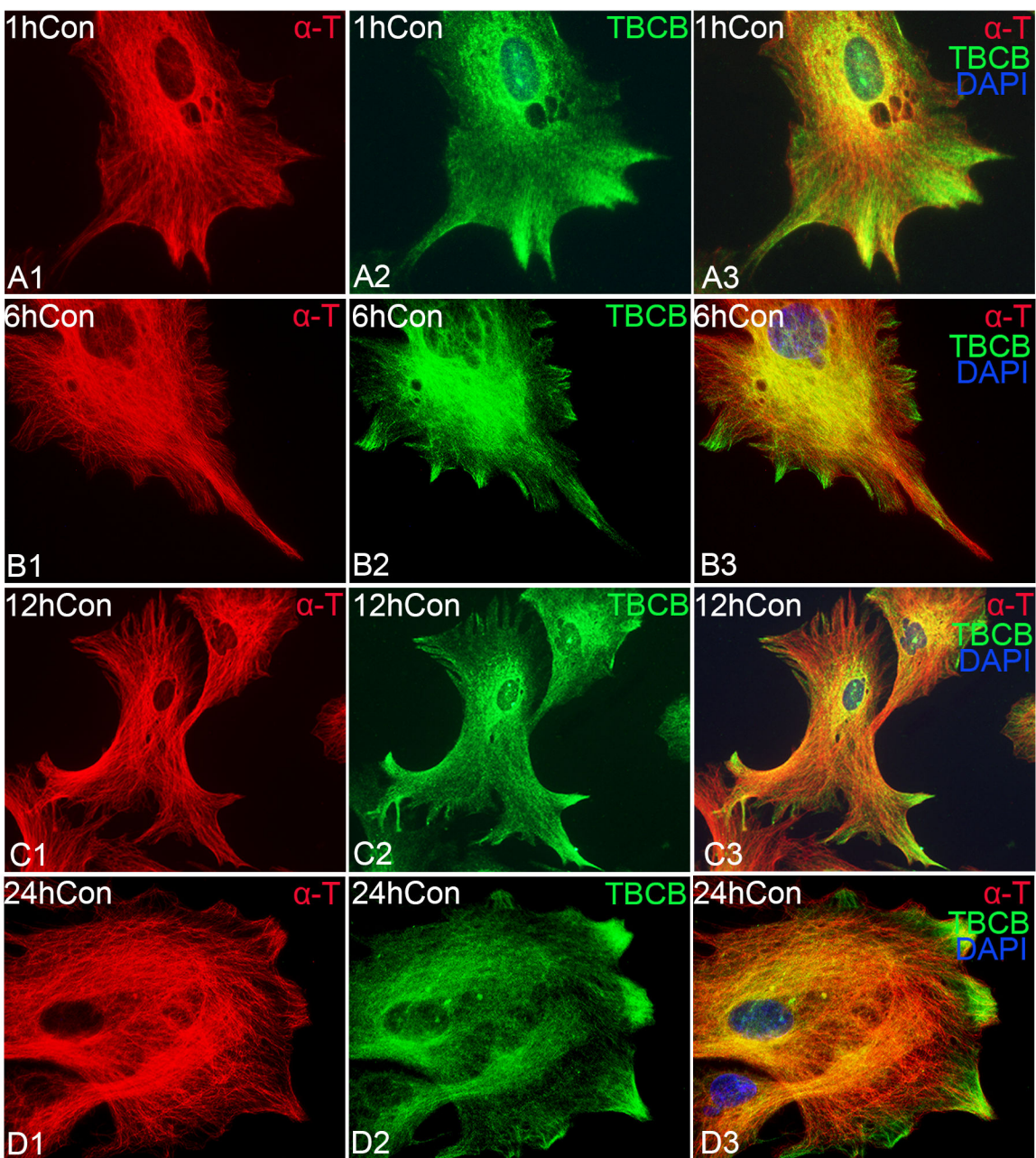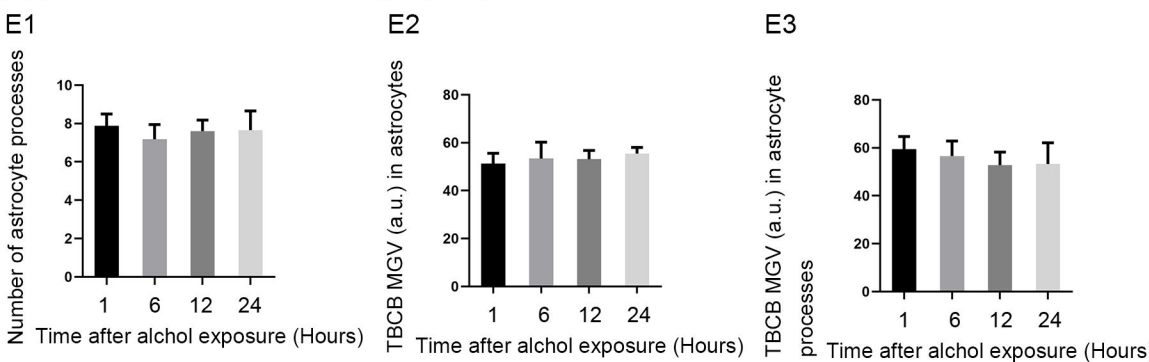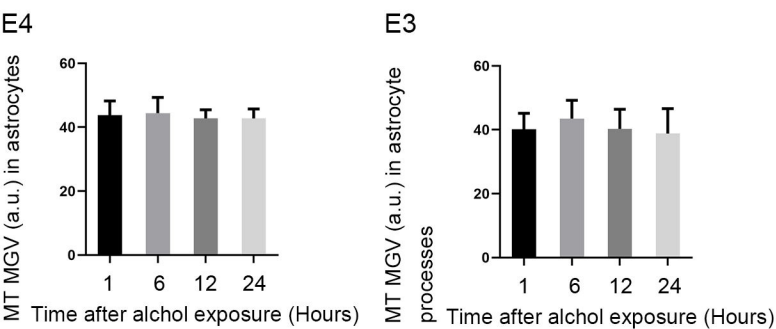

Supplement: Supplementary file 1 [file brainsci-12-00813-s001.zip › suppl.S2.pdf]
